# Characterization and Development of EST-SSR Markers Derived from Transcriptome of Yellow Catfish

**DOI:** 10.3390/molecules191016402

**Published:** 2014-10-13

**Authors:** Jin Zhang, Wenge Ma, Xiaomin Song, Qiaohong Lin, Jian-Fang Gui, Jie Mei

**Affiliations:** 1 Key Laboratory of Freshwater Animal Breeding, Ministry of Agriculture, Freshwater Aquaculture Collaborative Innovation Center of Hubei Province, College of Fisheries, Huazhong Agricultural University, Wuhan 430070, China; 2 State Key Laboratory of Freshwater Ecology and Biotechnology, Institute of Hydrobiology, Chinese Academy of Sciences, University of the Chinese Academy of Sciences, Wuhan 430072, China

**Keywords:** EST-SSRs, yellow catfish, 454 pyrosequencing, genetic diversity

## Abstract

Yellow catfish (*Pelteobagrus fulvidraco*) is one of the most important freshwater fish due to its delicious flesh and high nutritional value. However, lack of sufficient simple sequence repeat (SSR) markers has hampered the progress of genetic selection breeding and molecular research for yellow catfish. To this end, we aimed to develop and characterize polymorphic expressed sequence tag (EST)–SSRs from the 454 pyrosequencing transcriptome of yellow catfish. Totally, 82,794 potential EST-SSR markers were identified and distributed in the coding and non-coding regions. Di-nucleotide (53,933) is the most abundant motif type, and AC/GT, AAT/ATT, AAAT/ATTT are respective the most frequent di-, tri-, tetra-nucleotide repeats. We designed primer pairs for all of the identified EST-SSRs and randomly selected 300 of these pairs for further validation. Finally, 263 primer pairs were successfully amplified and 57 primer pairs were found to be consistently polymorphic when four populations of 48 individuals were tested. The number of alleles for the 57 loci ranged from 2 to 17, with an average of 8.23. The observed heterozygosity (*H_O_*), expected heterozygosity (*H_E_*), polymorphism information content (*PIC*) and fixation index (*FIS*) values ranged from 0.04 to 1.00, 0.12 to 0.92, 0.12 to 0.91 and −0.83 to 0.93, respectively. These EST-SSR markers generated in this study could greatly facilitate future studies of genetic diversity and molecular breeding in yellow catfish.

## 1. Introduction

Molecular marker systems, such as simple sequence repeats (SSRs) or microsatellites [1], single nucleotide polymorphism (SNPs) [2], amplified fragment length polymorphisms (AFLPs) [3] and random amplification of polymorphic DNAs (RAPDs) [4] have been developed and are applied to fisheries and aquaculture. Yellow catfish is an important freshwater fish for its delicious flesh and high market value, whereas overfishing is decreasing its number and genetic diversity [5]. Applying genomic tools in the selection of elite broodstock has the potential to improve the productivity and commercial value of this species. In populations of yellow catfish, males grow faster than females by two to three folds. For this reason, an all-male monosex population has been massively produced for commercial purpose [3,6,7]. However, genetic resources and suitable molecular markers are still scarce in yellow catfish.

SSRs are tandem repeating sequences of 1–6 nucleotides and distributed throughout vertebrate genomes [8]. Based on their locations, SSRs can be classified into genomic SSRs (gSSRs) and Expressed Sequence Tag-SSRs (EST-SSRs) [9]. Because of high level of polymorphism, SSRs have wide applications in population genetics, such as parentage analysis [10], Quantitative Trait Locus (QTL) mapping [11], marker assisted selection (MAS) [12], and phylogenetic studies [13]. Traditional methods of developing gSSR markers require fragmented genomic DNA and are usually time-consuming and labor-intensive. With the advent of high-throughput sequencing technology, the development of EST-SSRs has become a fast, efficient, and low-cost option for economical fish species [14,15].

The transcriptome of yellow catfish was acquired using a 454 GS-FLX Titanium platform and 540 Mbp of raw data were generated. In this study, we analyze the frequency and distribution of 82,794 potential EST-SSRs in the yellow catfish transcriptome. Sixty of 300 validated primer pairs were selected and further characterized for polymorphism analysis. Recently, we have performed genetic selection breeding on four wild populations of yellow catfish collected from Chang Lake (Jingzhou), Hong Lake (Honghu), South Lake (Zhongxiang) and Dongting Lake (Hunan) as previously reported [16]. These EST-SSR markers should provide a promising genetic resource for molecular breeding of yellow catfish.

## 2. Results and Discussion

### 2.1. Characterization of EST-SSRs in the Yellow Catfish Transcriptome

**Figure 1:**
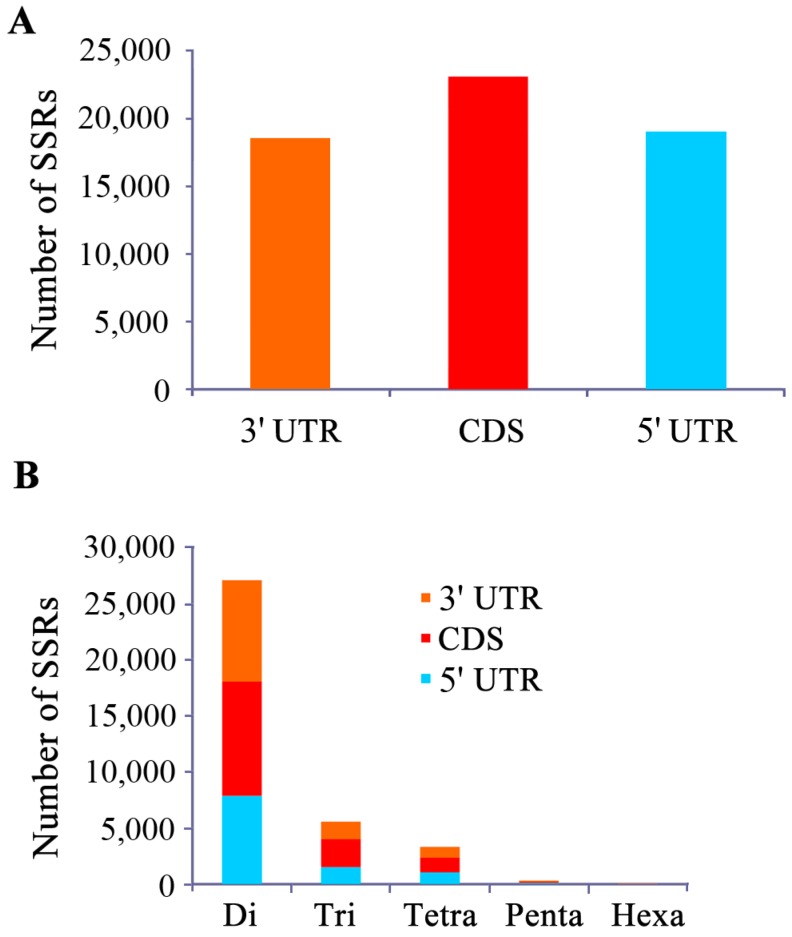
Distribution of EST-SSRs across the 5' UTR, CDS and 3' UTR in yellow catfish. Number of SSRs located on non-coding and coding region (**A**) and the distributions of SSRs with different motif sizes (**B**).

Putative open reading frames (ORFs) of all the assembled contigs and singletons were predicted by EMBOSS software. After analyzing the transcriptome by MISA software, we identified 82,794 SSRs, among which 23,085 SSRs (27.9%) are located in the coding region, 18,954 SSRs (22.9%) in the 5'-UTR, and 18,537 SSRs (22.4%) in the 3'-UTR (Figure 1A). Then, we analyzed the distribution of SSRs that have 2–6 bp repeat motif and are widely used. Of the 14,090 SSR identified in the coding region, dinucleotide accounts for 72.2% (10,180), tri-nucleotide is 17.6% (2478), tetra-nucleotide is 9.3% (1309), followed by penta-nucleotide 0.7% (98) and hexa-nucleotide 0.2% (25). Of the 10,584 SSR identified in the 5'-UTR, the most abundant is also dinucleotide accounting for 74.3% (7868), followed by tri-, tetra-, penta- and hexa-nucleotide with 14.5% (1532), 10% (1061), 1.1% (118) and 0.04% (5), respectively. Of the 11,654 SSR in the 3'-UTR, the percentage (and number) of di-, tri-, tetra-, penta- and hexa-nucleotide is 77.4% (9015), 13.4% (1559), 8.2% (961), 0.9% (107) and 0.1% (12), respectively (Figure 1B). Different locations of SSR markers in ESTs may suggest their possible for gene expression and functions [17]. The SSR insertions inside the promoter region of genes could modulate their expression levels [18].

**Figure 2:**
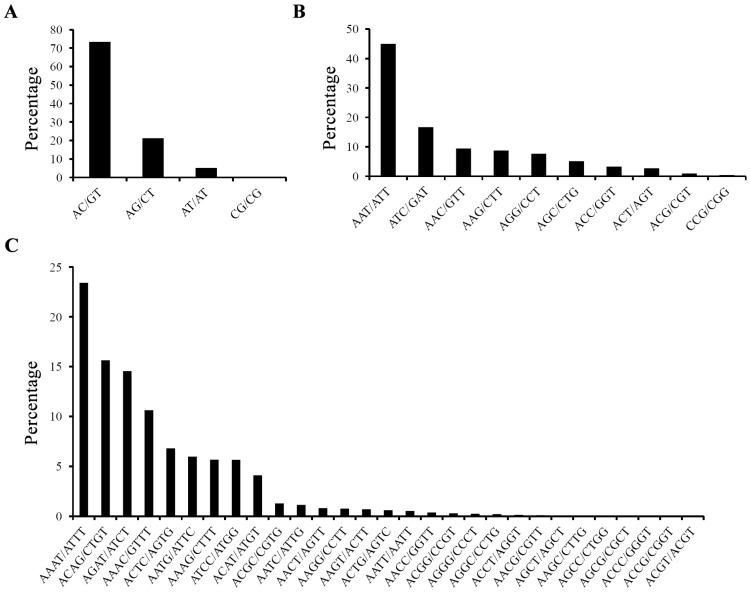
Characterization and frequency of different motifs among dinucleotide repeats (**A**), trinucleotide repeats (**B**) and the tetranucleotide repeats (**C**) EST-SSRs of yellow catfish.

Among the 82,794 SSRs, di-nucleotide is the most abundant type of repeat motif that is accounting for 65.14% (53,933) of the total SSRs, while hexa-nucleotide is the least type (84, 0.10%). Furthermore, the percentages of mono-, tri-, tetra-, and penta-nucleotide are 17.11% (14,168), 9.79% (8104), 7.28% (6027) and 0.58% (478) in respective. Most of SSRs had 6–36 repeat units, and six repeat units (15,004, 18.12%) and ten repeat units (9784, 11.82%) were the most represented types (Table 1). In the di-nucleotide repeat SSRs, AC/GT (39,554, 73.3%) and AG/CT (11,460, 21.2%) are the dominant types (Figure 2A). Similar to other fishes [19], (GC)n repeats are extremely rare in yellow catfish. Two most frequent repeats in the tri- nucleotide are AAT/ATT (3645, 45.0%) and ATC/GAT (1353, 16.7%) (Figure 2B). Among the tetra- nucleotide, the top two types of repeat motifs are AAAT/ATTT (1412, 23.4%) and ACAG/CTGT (943, 15.6%) (Figure 2C).

**Table 1:** Frequency of different repeat motifs among the EST-SSRs of yellow catfish.

Repeats	Mo	Di	Tri	Tetra	Penta	Hexa	Total	Percentage (%)
5	-	0	2654	1843	253	43	4793	5.79
6	-	12,561	1347	994	80	22	15,004	18.12
7	-	7110	893	632	44	8	8687	10.49
8	-	4411	537	421	16	5	5390	6.51
9	-	3248	384	316	18	3	3969	4.79
10	6769	2429	276	289	19	2	9784	11.82
11	3055	1972	263	225	15	0	5530	6.68
12	1805	1628	244	194	4	1	3876	4.68
13	995	1418	207	144	14	0	2778	3.36
14	602	1260	206	129	6	0	2203	2.66
15	392	1112	173	132	2	0	1811	2.19
16	174	1008	186	96	2	0	1466	1.77
17	136	896	141	110	1	0	1284	1.55
18	80	846	113	64	0	0	1103	1.33
19	53	806	128	60	3	0	1050	1.27
20	26	799	90	46	1	0	962	1.16
21	18	731	81	58	0	0	888	1.07
22	13	688	54	44	0	0	799	0.97
23	12	713	44	48	0	0	817	0.99
24	5	709	30	26	0	0	770	0.93
25	3	655	23	30	0	0	711	0.86
26	4	634	12	23	0	0	673	0.81
27	1	648	9	20	0	0	678	0.82
28	3	573	3	12	0	0	591	0.71
29	0	594	1	12	0	0	607	0.73
30	3	563	1	12	0	0	579	0.70
31	5	521	0	6	0	0	532	0.64
32	2	479	2	7	0	0	490	0.59
33	0	462	2	2	0	0	466	0.56
34	0	432	0	3	0	0	435	0.53
35	1	421	0	5	0	0	427	0.52
36	0	394	0	5	0	0	399	0.48
>36	11	3212	0	19	0	0	3242	3.92
**Total**	14,168	53,933	8104	6027	478	84	82,794	100.00
**Percentage (%)**	17.11	65.14	9.79	7.28	0.58	0.10	100.00	

### 2.2. SSR Marker Development and Genetic Diversity Analysis

A total of 300 SSR primers located on 280 assembled congtigs and singletons were randomly selected and amplified using DNA templates extracted from four wild populations of yellow catfish from Chang Lake, Hong Lake, South Lake and Dongting Lake. Of these SSR primers, 263 (87.7%) pairs of primers exhibited stable and repeatable amplification, and 57 (19%) of them were identified as polymorphic loci in all 48 individuals. Although we tried multiple PCR reactions under different amplification conditions, the 37 pair of primers still did not produce any PCR fragment, which probably due to assembly errors in sequences or primer pairs designed across a splice site with a large intron [20]. Among the 263 worked and 37 not-worked SSRs, there are 122 (46.4%) and 11 (29.7%) SSRs in the 3'-UTR, 71 (27.0%) and 12 (32.4%) SSRs in the 5'-UTR, 66 (25.1%) and 13 (35.1%) SSRs in the coding region, respectively. Further, there are 106 polymorphic and 157 unpolymorphic SSR markers, in which 41 (38.7%) and 81 (51.6%), 33 (31.1%) and 38 (24.2%), 30 (28.3%) and 36 (22.9%) SSRs were respectively located in the 3'-UTR, 5'-UTR and coding region. Moreover, tetra-nucleotide repeat is the most frequent form in both polymorphic SSRs (67.0%, 24 in the 3'-UTR, 21 in the 5'-UTR and 26 in the coding region) and unpolymorphic SSRs (51.6%, 36 in the 3'-UTR, 22 in the 5'-UTR and 23 in the coding region).

**Figure 3:**
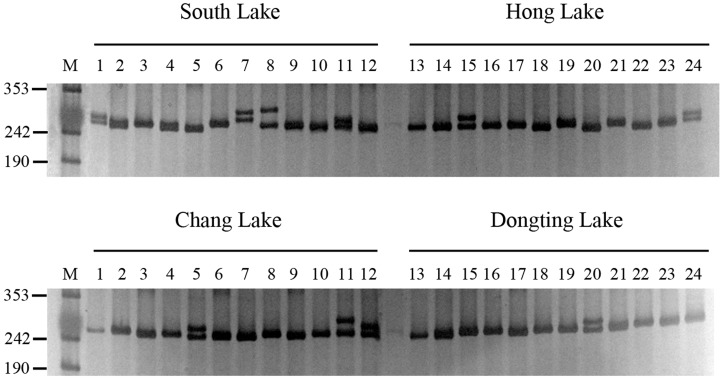
PCR amplification profiles of 48 yellow catfish accessions using primer pair H86. The PCR amplified products were separated on 7% polyacrylamide gel. M indicated the molecular markers.

A representative set of yellow catfish accessions amplified by primer pair H86 was shown in Figure 3. The selected 57 polymorphic primer pair sequences were characterized and deposited in GenBank to provide a foundation for breeding and genetic research of yellow catfish (Table 2).

Across the four populations of 48 individuals surveyed, the number of alleles (*N_A_*) per locus varied widely among the markers (Table 2) and ranged from 2 to 17, with an average of 8.23 alleles. We made an analysis of the observed (*Ho*) and expected heterozygosity (*H_E_*). The former value was ranged from 0.04 to 1.00 with an average of 0.52, while the latter varied from 0.12 to 0.92 with an average of 0.70. The high value of mean *Ho* and *H_E_* suggests that there is a relatively high heterozygosity. The overall polymorphic index content (PIC) values were ranged from 0.12 to 0.91 with an average of 0.66. According to the criterion previously described, three categories were defined as high (PIC > 0.5), moderate (0.25 < PIC < 0.5) and low (PIC < 0.25) [21,22]. So these 57 primers exhibited high levels of *PIC*. Lastly, the fixation index (*FIS*) values were ranged from −0.83 to 0.93 with an average of 0.25.

**Table 2:** Characteristics of the 57 EST-SSR markers for yellow catfish. Population genetic diversity analysis at 57 SSR loci was shown under the parameters: number of alleles per locus (*N_A_*), observed heterozygosity (*H_O_*), expected heterozygosity (*H_E_*), polymorphic information content (*PIC*) and fixation index (*FIS*).

EST-SSR	Repeat Motif	Primer Sequences (5'–3')	T a (°C)	Allele Size Range (bp)	Description of Putative Function	GenBank Accession No.	Heterozygosity				
							*N_A_*	*H_O_*	*H_E_*	*PIC*	*FIS*
H2	(AAT)13	F: CTTCCAGGGGGCTTCTAAGT	51	138–180	F-box and WD repeat containing protein 7	KM211716	7	0.604	0.831	0.80	0.266
		R: TGTTTGTCGTCGCTGTTCTC									
H6	(ATAG)16	F: TGTTGTAATCTCTCAATGAAGGTG	53	252–348	Transposable element Tc1 transposase	KM216910	13	0.729	0.865	0.84	0.148
		R: TGTTTGTGGAAACATAGACAGTGA									
H13	(GT)10	F: AGAGCTAGGCCAAACTGCTG	53	141–205	Calcium binding protein 39	KM236563	7	0.917	0.720	0.67	−0.286
		R: TCAGGAAGAACCAAAGCTGG									
H15	(CA)15	F: CTCGACCAGTCCTGAGCTTC	53	209–240	NF-kappa-B inhibitor beta	KM216912	5	0.271	0.565	0.47	0.515
		R: GTCATCATCAACGGACAACG									
H16	(CA)17	F: GAGAGACAGCGAGCCTCAGT	58	121–180	NEDD4–like E3 ubiquitin protein ligase WWP2	KM216871	16	1.000	0.924	0.91	−0.094
		R: CTAGGGCACCACACACTCCT									
H17	(TTA)14	F: ACCACCTCCGAGACACGC	57	110–172	Hypothetical protein	KM216905	7	0.500	0.815	0.78	0.380
		R: CACCACCTTCTAAATGAACATCA									
H20	(TTA)17	F: ATGTGTTTCCCACAGTGCAG	58	152–248	No significant match	KM216903	11	0.542	0.824	0.80	0.336
		R: CCGTCTTTGACCCAGATGTT									
H28	(TGGAGC)6	F: GGGGCCTCTTGGGTTATTTA	57	153–216	Gonadal-soma derived growth factor precursor	KM216886	7	0.375	0.725	0.68	0.477
		R: GTGCCAGCCTTGAAACTAGG									
H29	(TTTTA)7	F: GCCCTACAGCAGAGCTGAAC	57	102–132	Protein regulator of cytokinesis 1a	KM216864	4	0.417	0.550	0.47	0.234
		R: CGAGCAGAATCTCCTTCACC									
H32	(TGATGT)8	F: TTCGGGTAAAAAGTGATCCG	58	197–345	Predicted protein	KM216901	10	0.500	0.774	0.74	0.347
		R: CGAGAAGCGTTTAAAAAGGG									
H66	(AG)7	F: ATGGGATGACCAGGAGACAG	59	263–300	cAMP-dependent protein kinase catalytic subunit beta	KM236564	3	0.083	0.120	0.12	0.299
		R: GTCTTCCTCTCTGTGGCTCG									
H77	(TG)7	F: AAGCATAGATTTGCGCGTCT	58	264–334	Glucocorticoid receptor 2	KM216888	3	0.354	0.298	0.26	−0.201
		R: TCAGCTTGATGCCATTGTTC									
H78	(GTAT)9	F: GACCAAAGTGGATCGGACTC	62	273–378	Glucocorticoid receptor 2	KM216909	3	1.000	0.552	0.44	−0.829
		R: ATAACCCAGCATCCTGCATC									
H84	(AC)24	F: TGTAAAGGGGGAAAACCACA	58	202–284	Low density lipoprotein receptor	KM216916	7	1.000	0.837	0.81	−0.207
		R: GTGAGGGTGTTGCAGAGGTT									
H86	(TG)11tc(TG)8	F: CTCCTCCAGAGTGTCTTCGG	59	255–305	Adenylate cyclase type 5	KM216892	9	0.917	0.715	0.66	−0.297
		R: GTGGTCGATACCCAGAAGGA									
H89	(TGGA)5	F: AATGACAATAGGGTGCGGAG	59	269–339	No significant match	KM216896	3	0.208	0.194	0.18	−0.085
		R: TCTATCCATCAGTCCAGTCCG									
H96	(GAAT)5	F: GCACTCCGTCCAAGGTGTAT	59	173–181	No significant match	KM216857	2	0.292	0.252	0.22	−0.171
		R: TACCTGCCTGGTCAGTGTCA									
H106	(TTCT)5	F: TGATTTTTGGGACAGAGGAAA	59	202–264	No significant match	KM216856	14	0.604	0.903	0.88	0.324
		R: TCAAACTCAAAGTCAAAGGCAA									
H107	(TTCT)5	F: TGATTTTTGGGACAGAGGAAA	58	238–294	No significant match	KM216891	5	0.375	0.622	0.56	0.391
		R: TCAAACTCAAAGTCAAAGGCAA									
H109	(TTTTG)6	F: TATTTCCCTGTGGTGCTTCC	58	275–315	Heterogeneous nuclear ribonucleoprotein U protein 1	KM216875	13	0.417	0.908	0.89	0.537
		R: TTACGAAGCGTTCGAGTGTG									
H114	(TCTGT)5	F: TGAGGGGGTGCTAACTTTTG	59	215–322	Probable palmitoyltransferase ZDHHC20–like	KM216914	5	0.313	0.636	0.57	0.503
		R: GGAGGAACGAGAAACAGCAC									
H135	(ATCTA)5	F: GCATGACAGTGCTCGTTGTT	59	140–225	No significant match	KM216858	9	0.563	0.737	0.69	0.229
		R: TGAAAGTGGACGGTGACAAA									
H139	(TTAGC)6	F: GCTAGCGGCATTGTTAGCAT	58	154–204	Cyclin-dependent kinase 2 associated protein 2	KM216895	4	0.042	0.609	0.52	0.931
		R: CAAAAACCCACACACACTCG									
H147	(TCTA)25	F: TTGCCCAATTATACCACTTGC	58	229-264	Uncharacterized protein LOC101056656, partial	KM216859	14	0.563	0.818	0.79	0.305
		R: TCCAGCATTAAAATGAGGCAC									
H149	(ATCT)22	F: TTGCACTTATTGGGGATGTG	58	210–272	Hypothetical protein PANDA_009670	KM216860	11	0.604	0.790	0.76	0.227
		R: AACGGGAGGCTCTAACCAGT									
H151	(TGTT)11	F: CACTGATGATGGAATTGGGA	59	143–183	Glycogen phosphorylase, liver form	KM216904	5	0.438	0.711	0.65	0.378
		R: TCCCCTGCTCTGACAGTTTT									
H152	(AGTT)15	F: GAAACGGATATTTAGTGGGGG	59	191–252	No significant match	KM216879	10	0.771	0.868	0.84	0.102
		R: GCAATCACCAATAGAGCGAA									
H153	(ACAT)12	F: TGCCAGTATCTGACAACCCA	58	164–204	Collagen type IV alpha-3–binding protein-like	KM216898	8	0.625	0.762	0.72	0.172
		R: TTTTTAGTGGCCCATGTCTT									
H154	(TTTC)14	F: GAACTGTCCTTTGCTTTCGC	58	223–283	E3 ubiquitin-protein ligase MIB2	KM216861	17	0.604	0.924	0.91	0.339
		R: GTAGGGACTGACGATGGGAA									
H155	(AATA)15	F: CCTTTCTATTGTGCGTTGGC	59	232–344	No significant match	KM216862	11	0.604	0.857	0.83	0.288
		R: GGACATCGTAGCGAACTTCC									
H156	(AAAT)15	F: CATAACCGCACTGAATATGTGA	58	211–259	Family with sequence similarity 222, member B	KM216885	7	0.521	0.801	0.77	0.343
		R: AGCTGATTTTCAAGGCAGGA									
H158	(ATTT)16	F: ATCCATGCATCCTTCACACA	60	223–307	No significant match	KM216894	6	0.500	0.753	0.71	0.329
		R: ACATTCTGGCGTTTGGACTC									
H159	(ATCT)22	F: TTCATTGCTTAGTCTAGTTTACATC	58	217–332	No significant match	KM216893	4	0.271	0.613	0.55	0.554
		R: TCCTCAACCAGGTTAGTTACCA									
H160	(TTCT)11	F: CGTTGCACATTGGTGGTTTA	59	217–278	No significant match	KM216865	14	0.417	0.751	0.73	0.440
		R: TGGAGTGCAACAATGAGAGC									
H161	(CCAT)11	F: AGCAACAGTCGAGGAGCATA	59	161–202	Hypothetical protein PANDA_019388	KM216854	8	0.792	0.779	0.74	−0.027
		R: TGGTTGGGTGGATAGATGGT									
H163	(AAAT)11	F: GCCTTGATCAGCTTTCTTCC	58	286–382	No significant match	KM216884	4	0.583	0.659	0.59	0.106
		R: TGTTTGTAGGCCATGTCGAA									
H165	(CACT)11	F: GCGGAGACGCTTTCTGTATC	58	171–255	Muscle creatine kinase	KM216887	9	0.583	0.823	0.79	0.284
		R: AGGATGCAGCTGATTCAAGTC									
H166	(TGTT)11	F: AGCGTTAGCGTTAGCATCGT	58	157–233	Hypothetical protein ZEAMMB73_428483	KM216899	14	0.729	0.838	0.81	0.121
		R: ACACACAAACAGGAGCATGG									
H168	(ATCC)10	F: TGATCACGTGACCTCAGAGC	58	258–334	No significant match	KM216863	5	0.417	0.537	0.46	0.216
		R: TGATCACGTGACCTCAGAGC									
H169	(CATC)11	F: CGATCACATGTCACTCCTCC	58	221–292	Rho GTPase-activating protein 7–like	KM216906	7	0.563	0.805	0.77	0.294
		R: CATGCACTGGCACCCTAGTA									
H171	(ATAC)10	F: GATTCACCCAAAATGACATGG	58	173–248	Tribbles homolog 3	KM216872	10	0.271	0.492	0.48	0.444
		R: AAAGGCAATGACACTGCTCC									
H172	(AGAA)10	F: AGTGGTTCCGTTGAGGGTTT	58	255–328	No significant match	KM216913	6	0.500	0.762	0.72	0.337
		R: TTCTGACGTCTTCATGCTGC									
H176	(AATA)10	F: TGAAGGTCAGAAATGCAGAGC	58	118–145	No significant match	KM216876	5	0.833	0.761	0.71	−0.107
		R: CTGACCACGAAACAGCTGAA									
H203	(TGAT)8	F: CAGAGCCGGTGTTTCTTTTC	58	131–157	Protein LBH-like	KM216869	9	0.521	0.786	0.75	0.330
		R: CAGAACGCCTGTGCTGTTTA									
H216	(CTTT)8	F: GATGATGAGTTGCATGACGC	58	113–151	No significant match	KM216874	6	0.625	0.729	0.69	0.134
		R: TTTTTGTACGCACAGACCTGA									
H217	(ATTT)8	F: CTCGAATGGAAAAACCATCTG	58	231–257	No significant match	KM216908	5	0.458	0.656	0.59	0.294
		R: TTCCAGTGTACACGTTCACGA									
H228	(TTTA)8	F: CGGAGACGCTTAAGGACTTG	61	204–272	Zgc:63767 protein	KM216915	12	0.354	0.835	0.81	0.572
		R: GCTACAGATCAGAGCCCGTC									
H229	(ATTT)8	F: TTTTGCAAACGAATATCACCA	58	197–252	No significant match	KM216907	11	0.479	0.765	0.74	0.367
		R: CCCCCAACAACCTTGTTTAAT									
H233	(ATCA)8	F: CCACTCGGAAAGCTCAGAAC	58	244–286	No significant match	KM216890	8	0.229	0.497	0.47	0.534
		R: TACGTCGTTCCACAGCAGAG									
H237	(TCTT)8	F: TGGAGTAGTGCTGGTTCACG	58	248–301	No significant match	KM216880	12	0.458	0.841	0.82	0.449
		R: GAGAGAGAGCGACAGAGGGA									
H246	(ATA)9	F: GACGCAGCTCGTGAATGTTA	58	223–294	No significant match	KM216883	10	0.625	0.821	0.79	0.230
		R: AACCCTCACAAATCCCACAC									
H249	(ATT)13	F: GGGGAATAGTTATGAAAATGGG	58	276–326	No significant match	KM216877	9	0.229	0.684	0.62	0.662
		R: CACTCGCCTCCTAAAAGCAC									
H251	(AATG)9	F: CTGAGATAGGCACAGGCTCC	58	244–324	C1orf43–like protein	KM216866	9	0.375	0.656	0.63	0.423
		R: ACCCCGTTCAGTGTTGTCTC									
H254	(ATAA)8	F: TTCACTCAAATTCGTGTTCAAA	58	282–319	No significant match	KM216870	7	0.646	0.685	0.64	0.048
		R: TGTGGGGTGATTAGCATGAC									
H256	(GAAT)8	F: CAATGCACAAGCATGTAGGG	58	212–346	No significant match	KM216902	15	0.792	0.879	0.86	0.090
		R: CTGTAGGTGCCAAACTGCAT									
H259	(ATTT)12	F: CAGCATGGCCTTTCTTTGTT	56	263–326	No significant match	KM216853	8	0.333	0.613	0.59	0.451
		R: GGTTGCATGAGCAACTCAAA									
H260	(TCTG)17	F: GGATGTGGAGAGGCTTTGAA	58	218–248	No significant match	KM216855	6	0.208	0.620	0.55	0.660
		R: TCAGTCTCCATTACACTCCTGG									

## 3. Experimental Section

### 3.1. Fish Samples

Four wild populations of yellow catfish (2–3 years old) were collected from Chang Lake (Jingzhou), Hong Lake (Honghu), South Lake (Zhongxiang) and Dongting Lake (Hunan), as described previously [16]. 12 individuals were randomly selected from each population. Experimental protocols used here were approved by the institution animal care and use committee of Huazhong Agricultural University.

### 3.2. SSR Identification and Development of Primer Pairs

We have carried out 454 pyrosequencing technology to perform high-throughput deep sequencing of the yellow catfish transcriptome, with a cDNA library constructed by one RNA pool which has an equal quantity of total RNA extracted from ovary, testis, liver, kidney, muscle, brain, spleen and heart of yellow catfish (accession number of NCBI archive database: SRP032172). All types of SSRs from dinucleotides to hexanucleotides were identified from the assembled contigs and singletons using MISA software under default parameter settings: a minimum of ten repeats for dinucleotide SSRs, six repeats for dinucleotide SSRs, five repeats for trinucleotide, tetranucleotide pentanucleotide and hexanucleotide SSRs. Then we designed primers for the microsatellite sequences using the software Primer Premier 5.0.

### 3.3. Genomic DNA Extraction, PCR Amplification and Electrophoresis

Genomic DNA was extracted from the tail fin following the traditional proteinase K and phenol-chloroform extraction method, as described by Wang *et al*. [1]. The concentration of DNA was adjusted to 100 ng/μL, and DNA was stored at −20 °C until used.

To initially evaluate the polymorphism of the identified microsatellite markers, polymerase chain reaction (PCR) was performed using a 10 μL total volume that contained 0.5 mM each primer, 0.25μL each dNTP, 0.25 μL PCR buffer, 1 μL MgCl2, 0.5 units of Taq polymerase, and approximate 50 ng DNA. The following conditions were used for the PCR: 1 cycle of denaturation at 95 °C for 5 min and 35 cycles of 30 s at 94 °C, 30 s at a primer-specific annealing temperature, and 45 s at 72 °C. In the final step, the products were extended for 7 min at 72 °C. The PCR products were separated on 7% native polyacrylamide gel and visualized via silver staining. The allele size was estimated according to the pUC18 marker (TianGen Biotech, Beijing, China).

### 3.4. Evaluation of SSR Polymorphism and Genetic Diversity Analysis

To determine the polymorphism of these SSR loci, optimized primers were used to perform PCR reaction with genomic DNA extracted from 48 individuals of these four populations. PCR amplification was performed to accurately screen population-level variation, and PCR products were subjected to electrophoresis 7.0% non-denaturing polyacrylamide gels. To test the level of polymorphism at each EST–SSR locus in four populations , the number of observed alleles (*N_A_*), observed heterozygosities (*H_O_*) and expected heterozygosities (*H_E_*), fixation index (*FIS*) and polymorphism information content (*PIC*) values were calculated using popgene (Version 1.31) and CERVUS (Version 3.0.3).

## 4. Conclusions

By exploiting 454 transcriptome sequencing database, we obtained much information of EST-SSR makers. We not only developed 57 available EST-SSR makers, but also evaluated the population genetics of wild yellow catfish. This is the first report of a comprehensive study on the development and analysis of SSR markers by high-throughput sequencing in yellow catfish. Our results will provide a set of available EST-SSR markers that will be essential for future molecular breeding and genetic studies of yellow catfish.
